# Feeding behaviour of artificially reared Romane lambs

**DOI:** 10.1017/S1751731114000603

**Published:** 2014-03-25

**Authors:** I. David, F. Bouvier, E. Ricard, J. Ruesche, J.-L. Weisbecker

**Affiliations:** 1 INRA, UMR 1388 Génétique, Physiologie et Systèmes d’Elevage, F-31326 Castanet-Tolosan, France; 2 UMR 1388 Génétique, Physiologie et Systèmes d’Elevage, Université de Toulouse INPT ENSAT, F-31326 Castanet-Tolosan, France; 3 UMR 1388 Génétique, Physiologie et Systèmes d’Elevage, Université de Toulouse INPT ENVT, F-31076 Toulouse, France; 4 INRA, UE0332 Domaine de la Sapinière, F-18390 Osmoy, France; 5 INRA, UE0065 Domaine de Langlade, F-31450 Montgiscard, France

**Keywords:** lambs, feeding behaviour, artificial rearing

## Abstract

A consequence of increasing litter size in sheep is that a portion of the lambs have to be reared artificially. Detailed information about the pattern of milk consumption of artificially reared lambs would help improve their management. The purpose of this study is to describe the individual and group feeding behaviour of 94 Romane artificially reared lambs from 5 to 28 days of age using an electronic automatic lamb feeder. Animals were located in four pens of 8 to 15 lambs of similar age with one teat per pen. They were fed *ad libitum*. In our experimental situation (group rearing, continuous lightning) on average a lamb made 1.4±0.7 visits to the teat per meal and 9.5±3 meals per day. Mean meal duration was 247±158 s and the mean daily time spent feeding was 38±25 min. The mean quantity of milk intake was 176±132 ml per meal and 1.68±0.8 l per day. With age, the number of daily meals and their duration decreased while the quantity of milk consumed per meal and per day increased. Females tended to make more visits to the teat per meal and perform more meals per day but their milk consumption per meal was lower. The feed conversion ratio was 1.36±0.2. Synchrony in feeding (group meal) was estimated as the percentage of lambs that wanted to access the teat within the same short period (relative group meal size). On average 65% of lambs in the pen wanted to access the teat within the same period, but for 35% of group meals the relative group meal size was >90%. There was no consistency in the order in which lambs accessed the teat during a group meal. Our evaluation suggested that electronic automatic lamb feeders are tools that can provide, on a large scale, data describing the feeding behaviour of artificially reared lambs. It is then possible to study factors influencing these traits in order to improve the outcome of artificially reared lambs.

## Implications

During the last decade there has been an increase in the prolificacy of ewes which requires that a portion of the lambs will be separated from their mothers and reared artificially. In comparison with maternal rearing, artificial rearing is more labour intensive and frequently associated with higher lamb mortality. We have developed an electronic automatic lamb feeder that can provide, on a large number of individuals, data to describe the feeding behaviour of lambs. Analysis of such data will help to make recommendations to improve the management of artificially reared lambs.

## Introduction

The total weight of lambs weaned per ewe is an important parameter of production for the meat market. It has been shown that litter size is the trait of highest economic importance (Wolfova *et al.*, 2009). Nonetheless, because it is difficult for ewes to produce enough milk for more than two or three lambs, increasing the litter size of the ewes requires that a portion of the lambs will be separated from their mothers and reared artificially. On farms, the mortality of artificially reared lambs is generally higher than that of maternally reared lambs (Bonnot *et al.*, 2010) for several reasons: (1) artificially reared lambs are subject to both emotional and nutritional stress (Napolitano *et al.*, 1995) which can induce disease (Griffin, 1989), (2) technical problems are of greater impact and can lead to the death of lambs. Several studies have been published about the effect of artificial rearing on lamb welfare, growth performance and meat quality (Napolitano *et al.*, 2002; Emsen *et al.*, 2004; Bimczok *et al.*, 2005) but, to our knowledge and conversely to what has been performed in calves, none have provided a detailed report of the milk feeding behaviour of artificially reared lambs. Actually, because most calves are artificially reared, a large literature about their feeding behaviour exists in this species. Performances, behaviour and welfare of calves have been studied depending on the way in which they are offered milk (Appleby *et al.*, 2001; Jasper and Weary, 2002; Jensen and Budde, 2006; Jensen, 2009; Miller-Cushon *et al.*, 2013a) or concentrate and forage (Montoro *et al.*, 2013; Miller-Cushon *et al.*, 2013b), the type of milk replacer (Hill *et al.*, 2013), the type of housing (Jensen, 2004; O'Driscoll *et al.*, 2006; De Paula Vieira *et al.*, 2010; Faerevik *et al.*, 2010; Duve *et al.*, 2012), the health status of the animal (Borderas *et al.*, 2009b). As for cattle, detailed information about milk consumption and feed conversion efficiency in sheep would help to improve the management of artificially reared lambs. To reach these objectives it is necessary to obtain a large number of individual milk consumption profiles. In that case, automatic and continuous recording of feeding events is preferable to visual inspection of the feeding. Actually, visual inspection is appropriate if we are interested in a detailed description of the feeding behaviour but it is too time consuming to perform during a long period of time on many animals. Consequently, the National Institute of Agronomical Research (INRA) has developed an electronic automatic lamb feeder that recorded the milk consumption for individual lambs. The objective of the study was, using the data provided by the electronic feeder, to describe the individual and group feeding behaviour of lambs in the particular situation of the experimental design and to analyse if lambs characteristics (i.e. sex, litter size, weight at birth) have an influence on feeding traits.

## Material and methods

Experiments were conducted on Langlade experimental farm (INRA-FRANCE) on Romane lambs born in November 2010 (60 lambs) and September 2011 (47 lambs). Lambs were born following a specific mating procedure performed for another experiment described in detail by David *et al.* (2013). Briefly, dams and sires were selected based on their genetic effects on the average daily gain (ADG) of lambs from 0 to 45 days. Three groups of dams (low, medium and high maternal genetic effects) and two groups of sires (low and high direct genetic effects) were thus identified. Dams and sires were mated in 3 by 2 factorial plans. Supernumerary (>2) lambs born from these matings were randomly selected and removed from their mother 24 h after parturition to be artificially reared. From day 2 to day 3 after lambing, these lambs were altogether in a same pen where they were taught to suck from a milk feeder (Legrain, Foulbec, France). Light was on all day long in the nursery in order to keep a close watch on the young lambs during all the lambing period. At day 3, lambs that showed capacity to suck easily from the feeder were moved to other pens in the same nursery in four small groups of 8 to 15 lambs on slatted floors (in pens of 4.5 m^2^). Each lamb had an electronic ear tag. In these pens, lambs were fed using an automatic self-feed feeder TAPO/QUATRO IFS (Förster-Technik, Engen, Germany) with Solvor Perfo milk replacer (200 g/l, Bonilait Proteines, 86361 Chasseneuil du Poitou, France, 23.5% protein, 25% fat, 3.5% water, 2.2% minerals, 7.5% ash). Lambs had free access to milk which was prepared all day long except from 12.00 to 12.30 h and maintained at 42°C in a 1 l container. The standard recommendation for this type of feeder is 15 to 20 lambs per teat (Förster-technik, 1998). There was one teat per pen. A narrow corridor that ends with a wood board with a hole of the size of the lambs head was built in front of each teat. Thus, only one lamb could access the teat at a time. When a lamb went to the teat, its identification number was read by a radio frequency identification (RFID) reader with an antenna. The time (month/day/year-hour/minute/second) and the identification of the lamb were then recorded in a database. When the lamb had finished sucking and left the teat, the quantity of milk consumed by the lamb as well as the time it left the teat was recorded in the database. The accuracy of milk consumption measurements was ±4 g. To distribute milk, the Förster feeder necessitated a higher suction pressure than the Legrain feeder. The lambs adapted to this feeding technology within 1 day. Thus, the experiment started on day 5. Lambs were not offered concentrates during the study period (5 to 28 days). They were weighed at birth, and 15, 21 and 28 days after lambing. Weight records were used to calculate the ADG over the 0 to 15, 15 to 21 and 21 to 28 days period.

Given these data, our objective was to describe the milk intake (quantity, period, frequency) at the individual and group level as well as the feed conversion ratio (FCR) of lambs in the experimental situation.

In order to study the individual milk intake of lambs appropriately, rewarded visits to the teat needed be structured into meals. To do so, we applied the method proposed by Tolkamp and Kyriazakis (1999) which consisted of modelling the frequency distribution of log-transformed interval lengths between visits to the teat. This distribution was assumed to represent two populations: one of short intervals occurring within meals and the other of long intervals occurring between meals. Thus, we fitted a mixture of two Gaussians on the frequency distribution of log-transformed interval lengths between successive visits to the teat to determine the meal criterion (Tolkamp *et al.*, 1998). To take into account the period of the day where milk was not available (12.00 to 12.30 h); if a visit to the teat was performed during this period in between two rewarded visits, the corresponding interval length was considered as missing. The meal criterion is the interval length where the two Gaussians cross; which insure that the least number of intervals is misassigned (Tolkamp and Kyriazakis, 1999). Intervals lower than the criterion were considered as within meal intervals; otherwise they were considered as between meals. The records were then grouped into meals and six traits describing individual milk intake were defined (Table 1): (1) the number of feeding events (visits to the teat) per meal, (2) the number of meals per day, (3) the time spent feeding per meals, (4) the time spent feeding per day, (5) the quantity of milk consumed per meal, and (6) the quantity of milk consumed per day.

**Table 1:** List of feeding traits studied

	Formula for animal ( 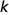 )
Individual feeding traits	
Number of feeding events per meal	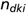
Number of meals per day	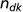
Time spent feeding per meal	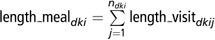
Time spent feeding per day	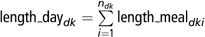
Milk quantity per meal	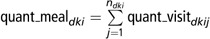
Milk quantity per day	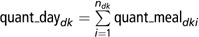
Feed conversion ratio (FCR)	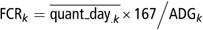
Group feeding traits	
Number of group-meals per day	
% of lambs accessing the teat during a group-meal	

Indices in the equations are animal *k*, day *d*, meal *i*, visit *j*. *n*_*dki*_ is the number of visits to the teat of animal *k* during its meal *i* of day *d*, *n*_*dk*_ is the number of meals of animal *k* during day *d*, 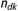 is the average quantity of milk drunk per day for animal *k*, ADG_*k*_ is the average daily gain for animal *k*.

Our objective was to identify factors influencing each trait and to estimate the trait repeatability (correlation between repeated measurements performed on the same animal). We also investigated if repeatability was linked to the growth of the lamb, that is whether it differed depending on the lamb’s growth class (given the distribution of lambs weaning weight, lambs belonged either to the ‘normal’ growth class if they weighed at least 6 kg at 35 days; otherwise they were classed ‘abnormal’). Comparison of trait repeatability between these two growth classes was performed to confirm or contradict feedback from breeders suggesting that growth problems could due to erratic milk consumption (i.e. drinking too much milk one meal, then too little at the next meal and so on). To evaluate if the milk intake varied depending on several characteristics of the lambs, we analysed factors influencing milk consumption traits. Factors were selected and evaluated using generalized linear mixed models (identity link for the number of meals per day, the quantity of milk consumed per day, the logarithm of the milk quantity consumed per meal, and the logarithm of the time spent feeding per meal and per day; log link for the number of feeding events per meal). All fixed effects and one-way interactions of biological relevance included in the models were selected in a step-wise manner, using nested models that were compared with the likelihood ratio test. The following effects were tested (Table 2): litter size (four classes: 1, 2, 3, 4+), sex of the lamb (two classes: male, female), year, type of sire (two classes: low and high direct genetic effects), type of dam (three classes: low, moderate and high genetic maternal effects), weight at birth (four classes: <2.5, 2.5 to 3.5, 3.5 to 4.5, ⩾4.5 kg), pen (four classes), growth class (two classes: normal or abnormal, 26 lambs), number of lambs in a pen (three classes: 7, [8,9,10], >10). Absence of colinearity between factors was checked before analysis. A lamb effect was included as a random effect in the models. We also tested, by comparing models using a likelihood ratio test, for heterogeneity of the variance of the lamb effect and of the residual depending on the growth class (i.e. different repeatability). Furthermore, to evaluate the relationship between variables, we calculated the phenotypic correlations between individual feeding traits.

**Table 2:** List of fixed effects affecting feeding behaviour traits of artificially reared Romane lambs

	Number of feeding events per meal	Number of meals per day	Time spent feeding per meal	Time spent feeding per day	Milk quantity per meal	milk quantity per day	Feed conversion efficiency
Age	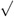	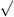	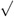	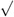	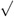	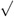	
Sex	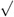	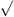			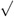		
Weight at birth							
Litter size							
Type of dam							
Type of sire							
Year	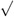	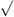	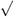	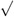		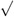	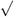
Pen	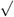	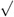	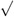		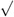	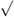	
Number. of lambs/pen	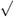	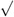		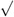	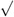	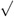	
Growth class		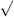		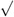	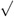	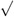	
Year×pen	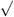					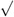	
Age×sex	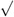						

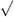
Significant effect with a risk *α* of 5%.

We calculated the FCR for lambs from the normal growth class as the ratio of the quantity of Solvor Perfo milk replacement powder ingested per day (i.e. the quantity of milk consumed per day (in l)×167) to the ADG for the three periods of age (0 to 15, 15 to 21 and 21 to 28 days). We selected the fixed effects influencing FCR in the same step-wise manner as previously described for individual feeding traits except that the lamb random effect was not included in the model.

Although the feeding behaviour of the lambs was not observed directly, the automatically recorded data was used to try to determine whether the animals in a pen competed and/or motivated each other when sucking. When a lamb accesses the teat; that could increase feeding motivation of the others or it can push away another lamb from the teat. In fact, feeding synchrony was observed previously (i.e. animals in a same pen usually stand up and want to access the teat during the same short period of time) and we would like to describe it. To analyse such group feeding, we structured the successive visits to the teat of the various lambs in each pen into group meals. Some visits were separated by relatively short intervals (the two lambs wanted to access the teat during the same short period of time), whereas others were separated by longer periods (between group meal intervals). The criterion defining a group meal was estimated by fitting three Gaussians on the frequency distribution of log-transformed interval lengths between the successive visits to the teat of the lambs in a same pen (time start visit_*n*+1_–time start visit_*n*_). The records were then gathered to form group meals. Motivation between animals was evaluated based on the percentage of lambs in a pen that went to the teat during the same group meal. Competition between animals was evaluated based on changes in individual meal duration and milk consumption depending on the ‘rank’ of the lamb to access the teat during a group meal. In order to do so, we defined four ranks: (1) the lamb accesses the teat first, (2) the lamb is in the first half of lambs (but not the first) to access the teat during this group meal, (3) the lamb is in the second half (but not the last), and (4) the lamb is the last to access the teat during a group meal. Finally, to determine whether lambs might be ‘leaders’ or ‘followers’, we assessed if lambs maintained the same rank by calculating rank repeatability and by visually examining changes in rank with time for each individual.

## Results

### Individual feeding behaviour

**Figure 1:**
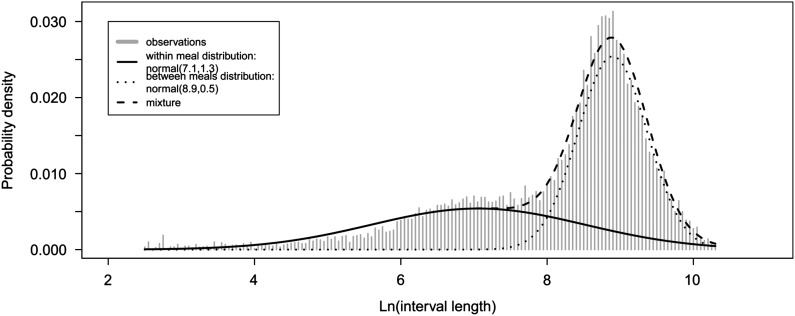
Fit of a probability density function consisting of a mixture of two Gaussian distributions on the log-transformed interval between successive suckling events of a lamb.

Twelve per cent of the lambs selected to be artificially reared died before 5 days of age and were not included in the study. 37 529 visits to the teat were registered for the 94 lambs having records between 5 and 28 days of age. Twenty-seven per cent of these visits did not involve milk intake; such visits were excluded from the analysis of individual feeding. Distribution of the logarithm of interval lengths between feeding events (Figure 1) showed that two types of intervals were observed: ‘within meal’ and ‘between meal’ intervals. The corresponding meal criterion was estimated at 2922 s (~49 min).

The mean number of feeding events per meal was 1.4, with a relative standard deviation (r.s.d.) of 50%; it did not vary a great deal with age (ranging from 1.35 to 1.55). Factors that significantly affected the number of feeding events per meal were the number of lambs in the pen, the combinations age×sex of the lamb and year×pen. On average, the number of feeding events per meal was higher for females than for males (+0.14), but the difference between sexes decreased with age. We observed that the number of feeding events per meal decreased with the number of lambs in the pen (−0.10 between extreme classes).

**Figure 2:**
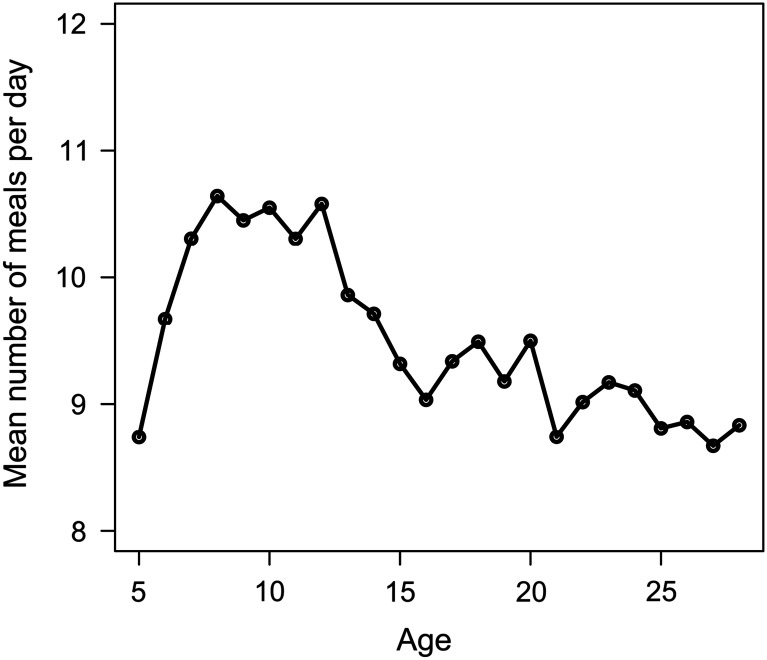
Change in the number of meals per day with age.

The mean daily number of meals per lamb was 9.5 (r.s.d.=30%) over the 5 to 28 days period. Changes to the number of meals per day with age are presented in Figure 2. The number of meals per day changed considerably over the 5 to 16 days period with an important increase then a decrease (maximum at 8 days of age). From then on the number of meals per day decreased slowly with age. The inter-individual variability of the number of meals per day was fairly stable with age (r.s.d. ranging from 0.23 to 0.33). Fixed effects that significantly affected the number of meals per day were the age of the lamb, the sex, the growth class, the number of lambs in the pen, the year and the pen. The number of meals per day was slightly higher for females than for males (+0.76), and for ‘normal growth’ lambs than for ‘abnormal growth’ lambs (+1.12). We observed that the number of daily meals increased then decreased when the number of lambs in the pen increased (+0.61 from the 1st [7] to the 2nd class [8,9,10], −1.09 from the 2nd to the 3rd class (>10)).

On average the time spent feeding per meal was 247 s (4 min 7 s) with a r.s.d. of 64%. It decreased linearly with age. After correction for the other factors of variation, the length of meals decreased significantly by 1 min 22 s between 5 and 28 days of age.

The mean time spent feeding was 2304 s per day (38 min 25 s) with a r.s.d. of 50%. Based on raw data, we observed a 35% decrease of this trait with age. Factors that significantly affected the time spent feeding per day were the year, age, number of lambs in the pen and the type of growth. Lambs from the abnormal growth class spent less time feeding (LS mean=33 min 39) than lambs from the normal growth class (LS mean=38 min 29). We observed a reduction of 3 min in the mean time spent feeding between extreme classes of the number of lambs in the pen.

The mean quantity of milk consumed per meal was 176 ml (r.s.d.=0.7). It increased with age (114 ml on day 5, 230 ml on day 28). The r.s.d. of the mean quantity of milk consumed per meal was high at all ages indicating an important variability of the trait between animals and meals. Nonetheless, this relative variability tended to decrease with age. Factors that significantly affected the quantity of milk consumed per meal were the age, sex, pen, number of lambs in the pen and growth class. The quantity of milk consumed per meal by lambs from the abnormal growth class was more than two times lower than for normal lambs. The quantity of milk consumed per meal was higher for males than females (+38 ml). It varied a little with the number of lambs in the pen (+16 ml for pens with <8 lambs in comparison with others).

The mean consumption of milk per day was 1.68 l (r.s.d.=0.5) and ranged from 241 ml to 2.88 l depending on the age and lamb. It increased with age with averages of 1 l on day 5 and 2 l on day 28, while the r.s.d. at a given age tended to decrease. Factors that significantly affected the quantity of milk consumed per day were the age, the combination year×pen, the number of lambs in a pen and the growth class. Lambs from the abnormal growth class consumed 1 l of milk less per day than lambs from the normal growth class. There was a decrease of milk consumed with the number of lambs in the pen (−52 ml from the 1st (7) to the 2nd class ([8 to 10]),.−141ml from the 2nd to the 3rd class (>10)). After correction for the other factors of variation, males consumed on average 161 ml more milk per day than females but the difference was not significant. Females took more meals per day than males that compensated for the smaller quantity of milk consumed per meal. Consequently, the daily milk intake was similar in males and females.

Repeatabilities are presented in Table 3. It is quite interesting to notice that, conversely to the traits observed at the level of the day, traits observed at the level of the meal (milk quantity, number of visits per meal and meal duration) had a very low repeatability. The repeatability of the number of meals per day was moderate, higher for lambs from the abnormal growth class (0.54) than for normal lambs (0.35). This results from the much higher inter-individual variation observed in abnormal growth lambs compared with normal growth lambs. The within-individual variance was also higher in abnormal *v*. normal growth lambs, but to a lesser extent. Repeatability of the daily time spent feeding was also moderate (0.27, 0.43 for lambs with an abnormal and normal growth, respectively). In comparison with the other traits, the repeatability of the quantity of milk consumed per day is quite high: 0.70 and 0.55 for lambs from the abnormal and normal growth classes, respectively. Except for the time spent feeding per day, traits at the level of the day were significantly less repeatable for lambs with a normal growth than for lambs with an abnormal growth.

**Table 3:** Repeatability (on the diagonal) and phenotypic correlations (upon the diagonal) among individual feeding behaviour traits of artificially reared Romane lambs

	Number of feeding events per meal	Number of meals per day	Length of meals	Time spent feeding per day	Milk quantity per meal	Milk quantity per day
Number of feeding events per meal	0.03/0.03^1^	0.22	0.58	0.29	0.07	−0.02
Number of meals per day		0.35 / 0.54	0.15	0.73	−0.21	0.23
Length of meals			0.09/0.09	0.47	0.19	−0.08
Time spent feeding per day				0.43/0.27	−0.23	0.02
Milk quantity consumed per meal					0.0 / 0.0	0.55
Milk quantity per day						0.55/0.70

Repeatability for normal (⩾6 kg at 35 days of age)/abnormal (<6 kg at 35 days of age) growth lambs.

1 The repeatability of *y* is calculated at the observed (original) scale: 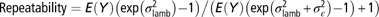 (Nakagawa and Schielzeth, 2010), where 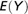 is the mean for 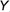;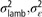 are the variances of the lamb and residual effect, respectively.

Phenotypic correlations (*ρ*) among the individual feeding behaviour variables are presented in Table 3. Results show that an increase in the number of visits to the teat per meal is associated with an increase of the meal duration (*ρ*=0.58) but not of the quantity of milk consumed per meal (*ρ*=0.07). We observed that when the quantity of milk consumed per meal decreased, the number of meals per day tended to increase (*ρ*=−0.21). Nonetheless, this increase does not compensate sufficiently for the lower amount of milk consumed per meal because the correlation between the milk intake per meal and per day are positively correlated (*ρ*=0.55). The time spent feeding per day is associated to a greater extent with the number of meals per day (*ρ*=0.73) than with the length of the meals (*ρ*=0.47).

The mean FCR was 1.36 (r.s.d.=0.15). The only factor that had a significant impact on FCR was the year the experiment was performed. Nonetheless, even although not statistically significant, we observed that, after correction for the year effect, the FCR of lambs from sires with high direct genetic effects was slightly lower (LS mean=1.37) than that of lambs from sires with low direct genetic effects (LS mean=1.39). The difference between the two values is obviously too small to draw any conclusions but the trend is as expected. From birth to 28 days of age, we observed that the increase in ADG is not related to a decrease of the FCR (Table 4), which indicates that it is a consequence of the increase of the milk consumption with age.

**Table 4:** Average daily gain (ADG) and feed conversion ratio (FCR) of artificially reared Romane lambs by period (standard deviation in bracket)

Period	ADG-artifical	FCR
0 to 15 days.	173 (101)	1.35 (0.2)
15 to 21 days	212 (103)	1.40 (0.2)
21 to 28 days	244 (87)	1.34 (0.2)

### Group feeding behaviour

**Figure 3:**
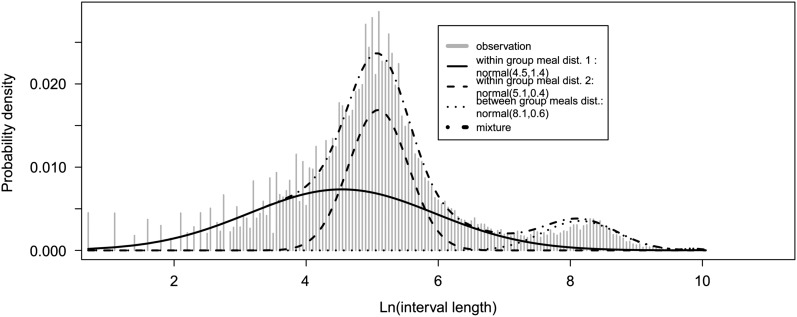
Fit of a probability density function consisting of a mixture of three Gaussian distributions on the log-transformed interval between successive visits to the teat of lambs in a pen.

The group meal criterion using the start–start intervals (time start visit_*n*+1_−time start visit_*n*_) was estimated at 1352 s (22 min 30 s) (Figure 3). 79% of the ‘stop–start time interval’ (time start visit_*n*+1_−time stop visit_*n*_) between visits during a group meal defined using this approach were 0 s. Given this meal criterion, the mean number of group meals per day was 17 (r.s.d.=16%). This number tended to slightly decrease with the mean age of the lambs in the pen. On average, group meals involved 65% of the lambs in a pen. For 35% of group meals, over 90% of the animals in a pen wanted to access the teat during the same short period of time. On the other hand, for 15% of the group meals only one lamb went to the teat. The lambs were not observed to suck preferentially at given periods during the day. We observed that, after correction for the other factors of variation, the milk consumption per visit tended to increase from rank 1 to rank 3 in a group meal. However, no clear trends were observed as to the duration of visits depending on the rank of the lamb (Table 5). The repeatability of the rank of the lambs in group meals is extremely low (<0.02). No lambs could be identified as being preferably ‘leaders’ or ‘followers’. We noted that lambs from the abnormal growth class tended to have a higher rank than normal lambs. Furthermore, the rank of females tended to lower than that of males.

**Table 5:** Effect (LSmeans (s.e.)) of the rank in a group meal on the quantity of milk consumed per visit and on the length of individual visits of artificially reared Romane lambs

Rank (1951 group meals)	Milk quantity (ml)	Length of visit (s)
1	65 (3)	134 (2)
2	71 (2)	116 (1)
3	90 (2)	132 (1)
4	87 (3)	216 (2)

## Discussion

The mortality observed before 5 days of age was lower than that reported for artificial reared Romane lambs (31%; François, 2012) and for other breeds in the experimental farm of Langlade (18%; Weisbecker, 2009). Average growth rate was in line with that reported for the breed (French national recording scheme).

### Individual feeding behaviour

The proportion of unrewarded visits (27%) is considerably different from the 79% reported by Bimczok *et al.* (2005); this is due to different feeding schemes as lambs were not fed *ad libitum* in their experiment. Similar differences in the proportion of unrewarded visits between animals fed restricted quantities of milk or *ad libitum* have been reported in other species (De Paula Vieira *et al.*, 2008). Unrewarded visits have been reported as an indication of hunger in several studies (De Paula Vieira *et al.*, 2008). Because the milk was available *ad libitum*, hunger was not the likely explanation for an unrewarded visit in the present study but corresponded to an animal that is ‘playing’ in front of the teat.

To estimate the meal criterion, we chose to fit a mixture of two normal distributions to the data. Other mixture of distributions have been proposed in the literature (Yeates *et al.*, 2001). In our case, the mixture of a weibull and normal distributions provided a less better fit to the log-interval distribution than the mixture of two normal (AIC=56 288 *v*. 56 053) and was less suitable to describe the observed probabilities of starting feeding. This result may be explain by the fact that, conversely to Yeates *et al.* (2001), we did not observed a linear relationship between the logarithm of the probability of starting a feeding and the logarithm of the time interval between rewarded visits to the teat. Unrewarded visits to teat were discarded from the analysis to estimate the individual meal criterion because part of the unrewarded visits of an animal corresponded to successive visits with time interval of 0 s between them. These 0 s intervals led to difficulties in the model fitting because it induced noise in the tail of the distribution. Anyway, the meal criterion estimated using all the visits to the teat is not very different than the one excluding unrewarded visits (47 min instead of 49 min). Our meal criterion is longer than meal criteria reported for other species: 2 to 8 min (calve), 6 min (beef cattle), 13 min (goat), 18 to 20 min (pig), 22 min (sheep), 30 to 50 min (cow) (Hammell *et al.*, 1988; Yeates *et al.*, 2001; Von Keyserlingk *et al.*, 2004; Gorgulu *et al.*, 2011 and 2012; Tolkamp *et al.*, 2011; Bailey *et al.*, 2012). Unfortunately, no reports are available on the meal criteria in pre-weaning lambs to compare with. The discrepancy between our meal criterion and those reported in other species maybe due to the specificity of lambs at this age which are monogastric and feed by drinking. Furthermore, as suggested by Tolkamp *et al.* (2011) for group housed pigs, the within meal interval might be overestimated due to group-housing which leads animals to compete to access the teat and induces sucking/licking behaviour. In accordance with this hypothesis and our observations, Von Keyserlingk *et al.* (2004) estimated a possible meal criterion at 40.7 min for group-housed dairy calves and described calf-calf contacts similar to behaviour of the lambs observed in our experiment.

The mean number of feeding events per meal was slightly lower than the two to five feeding events reported for other species (Azizi *et al.*, 2010). The number of meals per day was close to the one reported in adult sheep (8.94, Gorgulu *et al.*, 2011) and calves (≈10, Boe and Havrevoll, 1993; Appleby *et al.*, 2001; Von Keyserlingk *et al.*, 2004), but lower than that reported in maternally reared lambs (≈30, Ewbank, 1964; Hess *et al.*, 1974). Nonetheless, comparison is difficult since the latter two reported measured the number of suckling events per day and not the number of meals. The increase in the number of meals per day with age observed over the 5 to 8 days period probably reflected the adaptation of the lambs to the feeder and was not normal feeding behaviour, while the decrease observed after 8 days was similar to observations in calves (Borderas *et al.*, 2009a). Few reports have described differences of the number of meals per day between male and female animals, except in pigs. Indeed, Hyun *et al.* (1997) observed no differences between males and females but the number of meals per day was significantly higher in barrows, while De Haer and De Vries (1993) showed that, in accordance with our results, boars ate less frequently than gilts. The less frequent feeding of lambs from the abnormal growth class may be due to problems in adapting to the feeder. These less healthy lambs might not manage to feed correctly and their access to the teat could be limited due to competition with the other lambs. Unfortunately, no long-term direct observations of the feeding behaviour were performed to evaluate these hypotheses.

The time spent feeding per meal was much higher than that reported (22 to 44 s) for maternally reared lambs (Hess *et al.*, 1974) and two times lower than that reported for calves (Appleby *et al.*, 2001; Gorgulu *et al.*, 2012). The discrepancy between our findings and those of Hess *et al.* (1974) is due to the difference in the meal definition used. The mean time spent feeding per day was slightly higher than that observed in calves (Boe and Havrevoll, 1993) and maternally reared lambs (≈24 min) as determined by Akdag and Teke (2011). Decrease in the mean time spent feeding with the number of lambs in the pen was in line with that reported in calves (Jensen and Budde, 2006).

The mean quantity of milk consumed per meal was higher than the meal size (129 ml) reported for German Grey Health lambs by Bimczok *et al.* (2005). In pigs, De Haer and De Vries (1993) observed, in accordance with our results, that feed intake per visit was higher for males than for females. A similar increase of the quantity of milk consumed per day with age was observed in calves (Jasper and Weary, 2002; Borderas *et al.*, 2009a).

Conversely to expected, except for the time spent feeding per day, repeatability of the traits at the level of the day were significantly lower for lambs with a normal growth than for lambs with an abnormal growth indicating that probably an ‘erratic’ feeding behaviour is not linked to a growth problem. Nonetheless, such result needs to be confirmed.

The mean FCR was higher than that reported by Bimczok *et al.* (2005) in German Heath lambs but this could be due to the use of different milk replacer.

### Group feeding behaviour

To our knowledge, this is the first time that time interval between visits to the teat was used to define group meal and to study feeding competition and motivation. Usually competitive behaviours and motivation was assessed by video recording (Appleby *et al.*, 2001; Von Keyserlingk *et al.*, 2004; Nielsen *et al.*, 1995). In order to structure visits to the teat into group meals, rewarded and unrewarded visits to the teat were taken into account. It is important that animals that got near to the teat but did not have time to suckle because they were pushed away by other lambs be taken into account to describe group feeding behaviour. To structure the visits to the teat into group meal, we defined the time interval as start_time_*n*+1_–start_time_*n*_ (start–start time interval) between successive visits *n* and *n*+1 to the teat for the lambs of a given pen (conversely to time intervals studied for individual feeding behaviour, visits *n* and *n*+1 were usually not performed by the same lamb). This interval reflected the time a lamb had to wait before suckling given that it was the next in line to access the teat. Distribution of the logarithm of the ‘start–start time intervals’ seemed to be more adapted to define the group meal criterion than a time interval defined as start_time_*n*+1_–stop_time_*n*_ (stop–start time interval) similar to the one used to define individual meal criterion. Actually, the distribution of the stop–start time intervals demonstrated that an important proportion of time intervals (66%) lasted 0 s. Such a number of points at 0 s led to difficulties fitting the distributions on the logarithm of the time intervals between visits to the teat. Three normal distributions (Figure 3) were fitted to the distribution of the ‘start–start time intervals’. Two populations of intervals were defined within group meals. They corresponded to the two types of visits to the teat: rewarded or unrewarded visits. Actually, the mean time spent to the teat for unrewarded visits was lower than for rewarded visits (76 *v.* 159 s). This difference is in the same range as the one between the means of the two within group meal distributions (83 *v.* 174 s). Behavioural synchrony, especially feeding synchrony, has been described in many species such as poultry (Appleby *et al.*, 2004), pigs (Nielsen *et al.*, 1995) and sheep (Boissy and Dumont, 2002; Ramseyer *et al.*, 2009), but little is known about feeding synchrony in pre-weaning lambs. We observed that the number of lambs involved in a group meal tended to decrease slightly with the average age of the lambs in a pen. This may be a consequence of the decrease of the time spent feeding per day with age which increases the time the teat is free (no queuing). We could have expected to observe an increase of the relative size of the group meal due to an increase of the social interaction between lambs with the time spent together, as observed by Jorgensen *et al.* (2009) with ewes where the incidence of queuing at the feed barrier increased from 1 to 14 days after grouping.

The lambs were not observed to suck preferentially at given periods during the day. This is in accordance with Borderas *et al.* (2009a) who showed that calves fed *ad libitum* distributed their visits to the feeder throughout the day. Concerning the increase in milk intake with the rank of the lamb in a group meal; we can postulate that lambs that access the teat first are more likely to be disturbed during sucking by other queuing lambs than the lambs that feed later on, and this reduces the quantity of milk that the first feeders can consume. Nonetheless, it would be necessary to observe feeding behaviours directly and quantify the levels of disturbance to confirm this hypothesis. The rank of females tended to be lower than that of males. This may be more a consequence of the greater number of visits to the teat and meals per day than because of a higher competitive behaviour of the males.

## Conclusion

The electronic automatic lamb feeder used in this study is an interesting tool to study the feeding behaviour of lambs. As in other species; we showed that with age, the number of meals per day decreased, as did the time spent feeding per day, while the quantity of milk consumed per meal and per day increased demonstrating that the intake capacity and speed of food intake increased with age. Our results show that when animals are fed *ad libitum* the feeding behaviour of males and females is different. Females tended to consume less milk per meal than males but took more meals per day than males. Consequently, the daily milk intake was similar for both males and females. Analyses performed in this study correspond to a description of the feeding behaviour of the lambs and is a starting point for studying management methods that will improve the feeding of artificially reared lambs. At this step of the analysis, we did not highlight any interesting factors that may help in the choice of the lambs to be artificially reared since the weight at birth and the litter size did not have a significant influence on feeding behaviour traits. Other characteristics of the lamb at birth will be interesting to study such as the vitality score of the lamb.
